# Probiotics-loaded nanoparticles attenuated colon inflammation, oxidative stress, and apoptosis in colitis

**DOI:** 10.1038/s41598-022-08915-5

**Published:** 2022-03-24

**Authors:** Abdullah. Glil Alkushi, Ahmed Abdelfattah-Hassan, Haitham Eldoumani, Sara T. Elazab, Sally A. M. Mohamed, Aya Sh. Metwally, Eman S.El-Shetry, Ayman A. Saleh, Naser A. ElSawy, Doaa Ibrahim

**Affiliations:** 1 Department of Human Anatomy, Faculty of Medicine, Umm Al-Qura University, Al Abdeyah, Mecca, Saudi Arabia; 2 Department of Anatomy and Embryology, Faculty of Veterinary Medicine, Zagazig University, Zagazig, 44511 Egypt; 3 Biomedical Sciences Program, University of Science and Technology, Zewail City of Science and Technology, October Gardens, 6th of October, 12578 Giza Egypt; 4 Department of Anatomy and Embryology, Faculty of Veterinary Medicine, Mansoura University, Mansoura, 35516 Egypt; 5 Department of Pharmacology, Faculty of Veterinary Medicine, Mansoura University, Mansoura, 35516 Egypt; 6 Department of Histology and Cytology, Faculty of Veterinary Medicine, Zagazig University, Zagazig, 44511 Egypt; 7 Department of Pharmacology, Faculty of Veterinary Medicine, Aswan University, Aswan, Egypt; 8 Department of Human Anatomy and Embryology, Faculty of Medicine, Zagazig University, Zagazig, Egypt; 9 Department of Animal Wealth Development, Veterinary Genetics and Genetic Engineering, Faculty of Veterinary Medicine, Zagazig University, Zagazig, 44519 Egypt; 10 Department of Anatomy and Embryology Faculty of Medicine, Zagazig University, Zagazig, Egypt; 11 Department of Nutrition and Clinical Nutrition, Faculty of Veterinary Medicine, Zagazig University, 1 Alzeraa Street, Zagazig, 44511 Sharkia Egypt

**Keywords:** Inflammatory bowel disease, Inflammation

## Abstract

Promising therapy is needed for treating inflammatory bowel diseases (IBD) to overcome current treatment that inefficient and associated with unnecessary health risks. Recently, the concept of incorporating natural products into nanocarriers has been intended as a promising therapy for treating IBD via modulating their stability and bioavailability. Thus, we aimed to explore the potential alleviating effects of dietary nano-supplement combined with bacillus strains (*Bacillus amyloliquefaciens*; BANPs) in colitis model. Rats were orally gavaged by 5% DSS and the efficacy and mechanistic actions of BANPs were evaluated by assessing the severity of clinical signs and inflammatory and apoptosis response, histopathological and immunohistochemistry examination in colonic tissues. The severity of clinical signs was successfully alleviated and fecal Lcn-2 levels, an important colitic marker, were decreased in BANPs then free BA treated groups. In contrast, inflammatory markers overexpression IL-6, IL-1β, TNFα, COX-2, and iNOS in the colitic group were reduced more prominently in BANPs treated group, unlike free BA. The amelioration of BANPs to colon injury was also correlated with oxidative stress suppression along with restoring total antioxidant capacity. Interestingly, BANPs treatment modulated apoptotic markers as proved by downregulation of cytochrome c, and caspase-3 and upregulation of Bcl-2 and Bax than free BA. The severity of the histopathological alterations in the colon was greatly reduced in BANPs than free BA groups. Remarkably, over-expression of ki67 and IL-6 in colonic tissues were suppressed in BANPs group. These findings together highlighted the beneficial efficacy of BANPs in IBD treatment which are evidenced by colonic inflammation alleviation. Taken together, these results recommend that BANPs is a promising agent that encourages its possible therapeutic role in colitis treatment.

## Introduction

Inflammatory bowel disease (IBD), involving of crohn’s disease and ulcerative colitis covers a group of pathological entities which is a major health problem in western and developed countries^1^. There are millions of patients with IBD worldwide with highest prevalence of young adults^2^. The etiology of IBD is a combination of key factors: genetic, immunological and environment factors such as a modern lifestyle, dietary habits, antibiotic routine and hygiene. These factors mainly alter the composition and diversity of intestinal microbiota and increase immune system stimulation thus controlling the progression and development of IBD^3^. Up till now, the disease is still regarded as incurable and all the treatments are mainly targeting the inflammation symptoms^4^. Additionally, the existing pharmaceutical treatments are risky or unsuccessful for long-term use and are accompanied by adverse side effects, therefore, new natural alternative therapies that combine efficacy and safety for IBD are needed. This has encouraged the development of new treatments, and the use of alternative and complementary medicine resources in IBD patients. In this context, using of dietary natural components is considered as the most important regulatory factor of colitis. Additionally, both healthcare specialists and patients have an increasing interest to role of nutritional therapy in maintaining IBD remission^5^. As diet may influence intestinal inflammation through various biological mechanisms including antigen presentation, gut microbiome modifications, function of the mucosal immune system and epithelial barrier^6^. On the other hand, gut microbiota appears to play a critical role in IBD management, specifically *Lactobacillus* and *Bifidobacterium* species, which possess significant health-promoting and immunomodulatory properties, on attenuating dextran sodium sulfate (DSS) DSS-induced colitis^7^. However, the exact molecular mechanisms underlying these applications is still largely unknown and need more investigations. The complex interactions of diet, normal intestinal microbiota, and health have promoted the introduction of probiotics that employ beneficial impacts on the host^8^. Oral formulations targeting a localized effect within the GI tract can be used as a rational drug delivery design for IBD^9^. From this point, dietary probiotics can be considered as a potential therapeutic tool, which is the ingestion of non-pathogenic microorganisms, for modifying the gut microbiome, improving immune response in colitis patients and associated with alleviation of IBD symptoms^10^. Other promoting health benefits of probiotics include modulating the gene expression related to gut inflammation^11^. Also, induction of antimicrobial peptides synthesis and the production of heat shock protein^12^. Despite, many researchers are interesting in application of probiotics recently, the efficacy of molecular signals and pathways of probiotics during the active phase of IBD inflammation (induction of remission) and as prevention of IBD (maintenance of remission) remains scarce.

Especially, promising evidence has indicated that *Bacillus amyloliquefaciens* (BA) is beneficial for the amelioration of inflammation and diarrhea^13^. Recently, One study also described the favorable effects of BA on IBD as it attenuated loss of body weight of dextran sulfate sodium salt (DSS)-induced colitis animals, besides decreasing the expression levels of pro-inflammatory cytokines in colonic tissues^14^.

On the other hand, nanotechnology provides new dietary supplements for special medical purposes. Hence, engineering of novel dietary formulation based on nanoparticles (NPs) are increasingly added to food and these nano-formulations offer food industry with many new approaches for improving quality, bioavailability and safety of food and increase its stability, moreover, these NPs protect active ingredients against degradation^15^. The fate of NPs in the gastrointestinal tract differs greatly from that of larger particles due to their increased surface area, greater motion ability, and easily penetrate the biological barriers, including epithelium of intestinal cells^16^. Moreover, microencapsulation of probiotic bacteria in to chitosan and Na-alginate nano particles increased its resistant to gastric conditions (pH 2.0), thus improved its viability and function though gastro-intestinal conditions^17^. Additionally, encapsulated probiotic bacterial cells can survive better than free probiotic cells in gastrointestinal conditions^18^. Currently, naturally occurring NPs used for nutrient delivery however, the impact on both commensal and pathogenic microorganisms and gut immune system, the mechanisms behind these not fully understood^19^. Thus, in our study we prospected that the combination of probiotics with specific nanoparticles will augment their long-lasting function in prevention and treatment of IBD. Specially if these NPs have additional beneficial properties as modulators of gut immune response, or have antimicrobial effects, besides ensuring probiotics arrival to targeted sites and increasing their circulation time inside the body. The overall aim of this study is to synthesize new probiotics-based NPs that will outcome in better efficacy of probiotic therapy against IBD. Thus, DSS-induced colitis rat models were used in our experiments to prove the possibility of generating “natural” nanovectors from probiotics that mediate efficient in vivo therapy for ulcerative colitis in human.

## Results

### Assessment of *B. amyloliquefaciens* loaded nano particles viability in activated gastric juice and simulated intestinal juice

The results showed that *B. Amyloliquefaciens* loaded nano particles was more stable in stimulated then intestinal gastric juice than free bacillus. Amyloliquefaciens after 3 h incubation period Table 1. As free *B. Amyloliquefaciens* load reduced to 8.9 ± 0.23 × 10^9^ at 30 min after incubation to 8.1 ± 0.21 × 10^5^ after 120 min. Meanwhile, the load of *B. Amyloliquefaciens* loaded nano particles was reduced from 9.3 ± 0.19 × 10^9^ after 30 min incubation period and to 6.1 ± 0.20 × 10^8^ after 120 min incubation period.

**Table 1:** Cell survivability (log CFU/mL) of free and double coated nano particles *bacillus.*

	No. of survival cells (CFU/mL)			
	30 min	60 min	90 min	120 min
Free *bacillus. amyloliquefaciens*	8.9 ± 0.23 × 10^9^	9.1 ± 0.21 × 10^8^	5.3 ± 0.11 × 10^7^	8.1 ± 0.21 × 10^5^
*bacillus. Amyloliquefaciens double coated nano particles*	9.3 ± .19 × 10^9^	8.6 ± 0.16 × 10^9^	5.3 ± 0.16 × 10^9^	6.1 ± 0.20 × 10^8^

*Amyloliquefaciens* (BANPs) after treatment in activated gastric juice then activated intestinal juice with bile salt.

*SE* standard error. ^a,b,c^Means of the bars with different letters were significantly different among groups (P < 0.05).

### Assessment of clinical signs related to colonic inflammation

**Figure 1:**
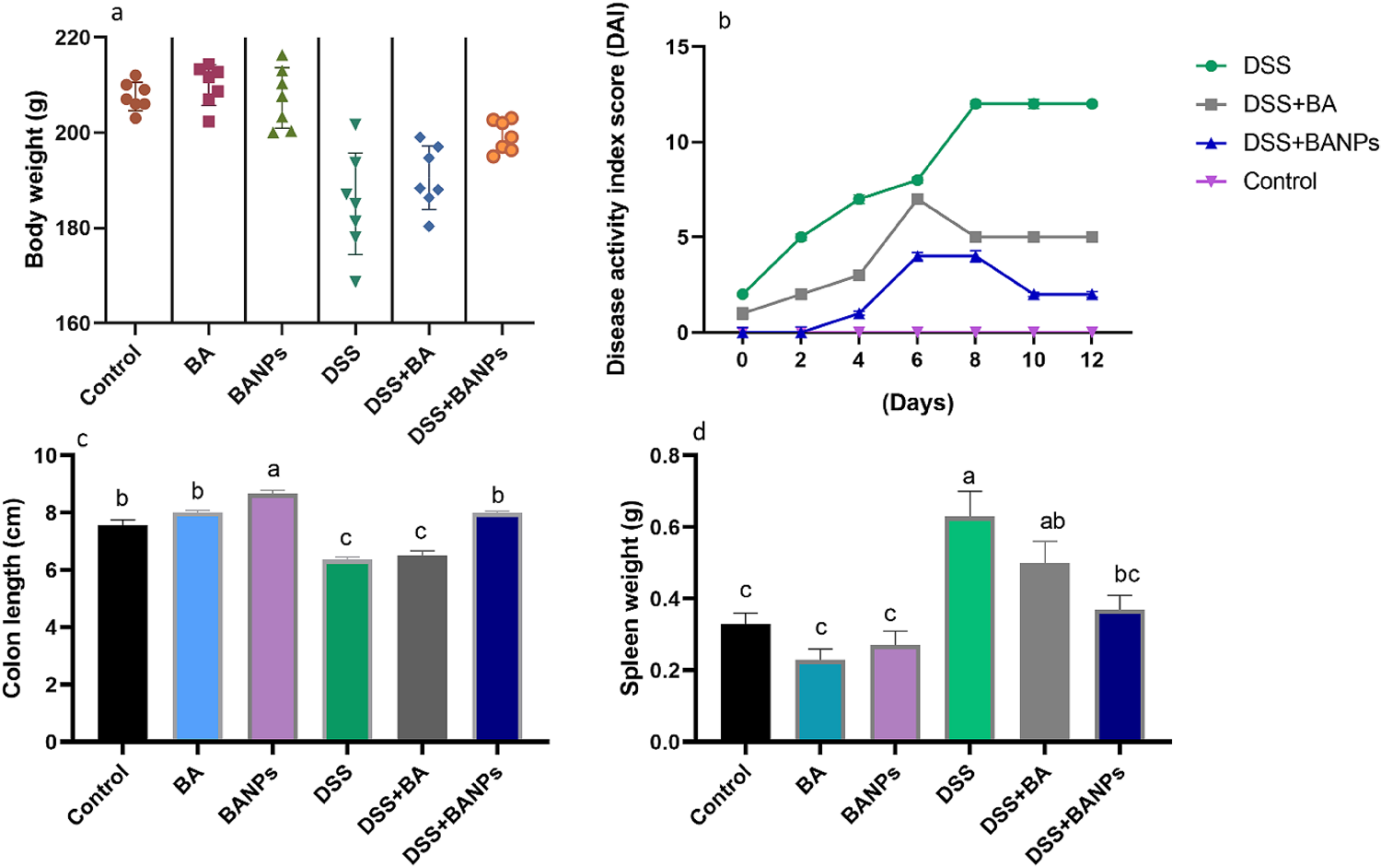
Effects of orally administered *B. amyloliquefaciens (BA) or BA-nanoparticles (BANPs)* in non-colitic and colitic Signs. (**a**) Body weight gain. (**b**) Disease activity index score. (**c**) Colon length. (**d**) Spleen weight. Non-colitic groups including Control, BA and BANPs groups where rats were orally gavaged PBS, *B. amyloliquefaciens and B. amyloliquefaciens* loaded nanoparticles (BA at level of 1.0 × 10^10^ CFU/kg in 1 mL of PBS/rat/day), respectively, for 7 days. Colitic groups: DSS, BA and BANPs groups where rats were orally gavaged dextrane sodium sulphate (DSS), *B. amyloliquefaciens* + *DSS and B. amyloliquefaciens* loaded nanoparticles (BA at level of 1.0 × 10^10^ CFU/kg in 1 mL of PBS/rat/day) + *DSS*, respectively, for 7 days. All groups orally gavaged by 5% DSS. Values are expressed as mean ± SE, ^a,b,c^Means of the bars with different letters were significantly different among groups (*P* < 0.05).

The efficacy of BANPs on signs of colitis for 14 days post induction in rats are presented in Fig. 1. Rats in DSS induced colitis suffered marked weight loss (up to 10%) as a result of colonic inflammation compared with 7% weight loss in BA treated group (Fig. 1a). Notably, faster recovery of body weight loss was detected in BA NPs treated group and with only 3% body weight loss (Fig. 1a). Progressive increase in DAI scores associated with incidence of diarrhea and rectal bleeding, a reliable marker of colon inflammation, was observed in DSS induced groups throughout the experimental period. Meanwhile, rats in BANPs treated group displayed the lowest significant DAI score and had a significantly longer colon (*P* < 0.05) when compared with DSS induced group (8.1 ± 0.40 vs 6.3 ± 0.22 cm, respectively) (Fig. 1b,c). Remarkable enlargement of spleen was found in DSS colitic rats, while group treated with BANPs exhibited spleen’s weight that nearly similar to non-colitic group (Fig. 1d). All rats treated with BANPs were alive at the end of the experiment whereas rats treated with free BA showed survival rate of 80% compared to colitic group of 50% survival rate.

### Hematological and biochemical estimation

Data concerning the hematological and liver and kidney function tests are illustrated in Table 2. Non-colitic groups showed significant (P < 0.05) higher levels of the RBCs counts and Hb concentrations than colitic groups. Additionally, administration of BA or BANPs had no significant effect on AST, ALT, urea and creatinine levels when compared with control group. Meanwhile, the counts of RBCs and Hb concentrations were reduced (P < 0.05) and indicators of liver and kidney function were distributed in DSS induced colitic group. Notably, orally administration of BANPs after colitis induction to large extent recovered liver and kidney function tests and kept RBCs counts and Hb concentrations in nearly the same as control non-colitic group.

**Table 2:** Hematological, liver and kidney function tests of non-colitic and colitic rats orally administered B. amyloliquefaciens (BA) or BA-nanoparticles (BANPs).

Parameter	Control	BA	BANPs	DSS	BA + DSS	BANPs + DSS	*P* value
RBCs (× 10^6^/μL)	10.40^a^ ± 0.76	10.90^a^ ± 0.09	10.89^a^ ± .54	6.98^c^ ± 0.36	8.70^b^ ± 0.44	10.40^a^ ± 0.45	< 0.001
Hb (g/dL)	11.93^b^ ± 0.1	12.27^a^ ± 0.25	12.53^a^ ± 0.21	7.96^d^ ± 0.09	9.40^d^ ± 0.19	11.50^c^ ± 0.14	< 0.001
ALT (U/L)	51.03^c^ ± 0.76	50.22^c^ ± 0.66	50.67^c^ ± 0.31	83.18^a^ ± 0.9	55.60^b^ ± 0.75	51.30^c^ ± 0.65	0.02
AST (U/L)	27.20^c^ ± 0.43	25.80^c^ ± .023	25.42^c^ ± 0.41	41.85^a^ ± 0.97	31.78^b^ ± 0.85	27.40^c^ ± 0.58	0.03
Urea	34.46^ cd^ ± 0.59	32.56^d^ ± 0.58	33.47^ cd^ ± 0.67	63.21^a^ ± .0.89	50.66^b^ ± 0.6	37.33^c^ ± 0.5	0.04
Creatinine	1.18^c^ ± 0.10	1.25^c^ ± 0.08	1.31^c^ ± 0.09	2.4^a^ ± 0.06	2.02^b^ ± 0.03	1.49^c^ ± 0.07	0.02

Non-colitic groups including Control, BA and BANPs groups where rats were orally gavaged PBS, *B. amyloliquefaciens* and *B. amyloliquefaciens* loaded nanoparticles (BA at level of 1.0 × 10^10^ CFU/kg in 1 mL of PBS/rat/day), respectively, for 7 days. Colitic groups: DSS, BA and BANPs groups where rats were orally gavaged dextrane sodium sulphate (DSS), *B. amyloliquefaciens* + DSS and *B. amyloliquefaciens* loaded nanoparticles (BA at level of 1.0 × 10^10^ CFU/kg in 1 mL of PBS/rat/day) + DSS, respectively, for 7 days. All groups orally gavaged by 5% DSS.

*RBCs* red blood cells, *Ht* hematocrit, *Hb* hemoglobin, mean values with different letters in the same row differ significantly at *P* < 0.05, *SE* standard error. ^a,b,c^Means of the bars with different letters were significantly different among groups (P < 0.05).

### Assessment of fecal lipocalin-2 levels

**Figure 2:**
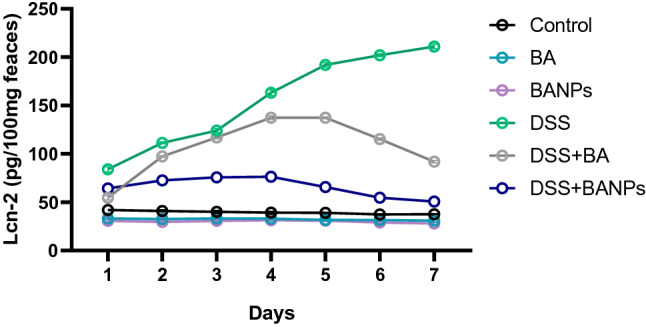
Effects of orally administered *B. amyloliquefaciens (BA) or BA-nanoparticles (BANPs)* in non-colitic and colitic rats on fecal lipocalin-2 (Lcn-2) levels 7 days post DSS induction. Non-colitic groups including Control, BA and BANPs groups where rats were orally gavaged PBS, *B. amyloliquefaciens and B. amyloliquefaciens* loaded nanoparticles (BA at level of 1.0 × 10^10^ CFU/kg in 1 mL of PBS/rat/day), respectively, for 7 days. Colitic groups: DSS, BA and BANPs groups where rats were orally gavaged dextrane sodium sulphate (DSS), *B. amyloliquefaciens* + *DSS and B. amyloliquefaciens* loaded nanoparticles (BA at level of 1.0 × 10^10^ CFU/kg in 1 mL of PBS/rat/day) + *DSS*, respectively, for 7 days. All groups orally gavaged by 5% DSS. Values are expressed as mean ± SE, ^a,b,c^Means of the bars with different letters were significantly different among groups (*P* < 0.05).

Levels of Lcn-2 in fecal samples obtained daily throughout treatment are presented (Fig. 2). Fecal Lcn-2 level in colitic group was increased from day 3 to day 10, while in group treated with BA its level was decreased from day 7 but did not reach the level of Fecal Lcn-2 in control non colitic group at day 10. Notably, Fecal Lcn-2 level was significantly decreased (P < 0.05) from day 6 and at day 10 its level was restored and reached to Lcn-2 level of control non colitic group.

### Assessment of MPO, NO in colonic tissues and CRP levels in serum

**Figure 3:**
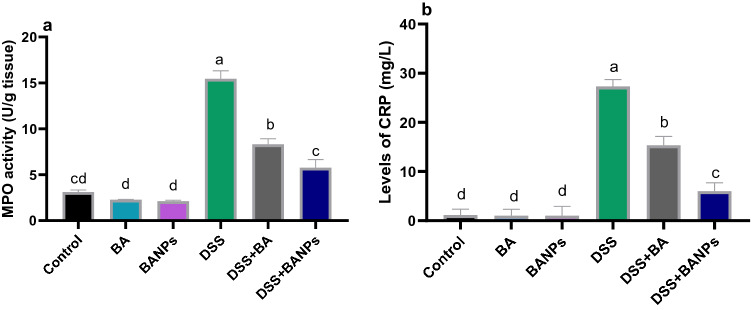
Effects of orally administered *B. amyloliquefaciens *(*BA*)* or BA-nanoparticles (BANPs)* in non-colitic and colitic rats on colonic myeloperoxidase (MPO) activity activity (**a**) and serum C-reactive protein (CRP) levels 7 days post DSS induction. Non-colitic groups including Control, BA and BANPs groups where rats were orally gavaged PBS, *B. amyloliquefaciens and B. amyloliquefaciens* loaded nanoparticles (BA at level of 1.0 × 10^10^ CFU/kg in 1 mL of PBS/rat/day), respectively, for 7 days. Colitic groups: DSS, BA and BANPs groups where rats were orally gavaged dextrane sodium sulphate (DSS), *B. amyloliquefaciens* + *DSS and B. amyloliquefaciens* loaded nanoparticles (BA at level of 1.0 × 10^10^ CFU/kg in 1 mL of PBS/rat/day) + *DSS*, respectively, for 7 days. All groups orally gavaged by 5% DSS. Values are expressed as mean ± SE, ^a,b,c^Means of the bars with different letters were significantly different among groups (*P* < 0.05).

The activities of MPO were elevated after induction of DSS to 15.47 and only for 3.9 in 8.3 U/g in groups received free BA and BANPs respectively, when compared with non-colitic group (3.12 U/g) (Fig. 3a). The highest levels (P < 0.05) of CRP in colitic group were alleviated in BANPs group followed by free BA group when compared to colitic group non treated group (Fig. 3b).

### Detection of oxidative stress and antioxidant defense in colon

**Figure 4:**
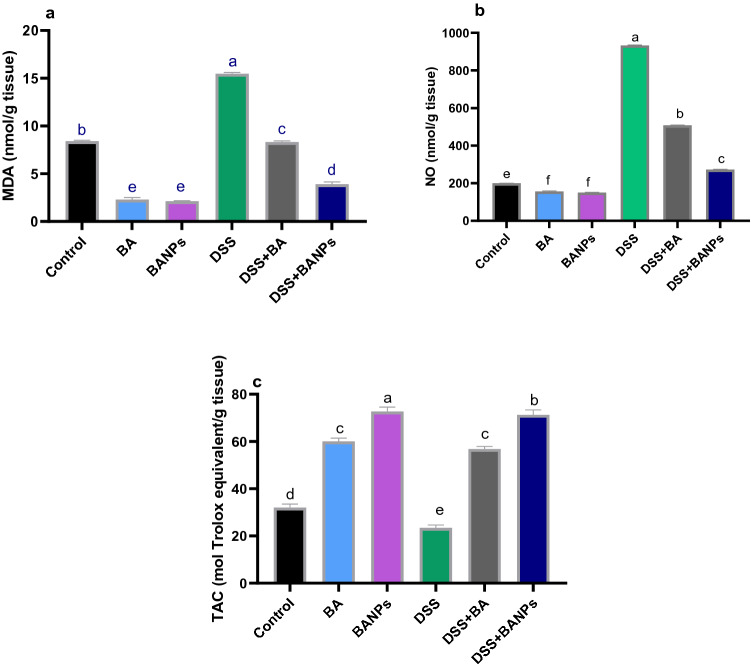
Effects of orally administered *B. amyloliquefaciens (BA) or BA-nanoparticles (BANPs)* in non-colitic and colitic rats on lipid peroxides expressed as malondialdehyde; MDA. (**a**) Nitric oxide; NO. (**b**) Total antioxidant capacity; TAC (**c**) 7 days post DSS induction. Non-colitic groups including Control, BA and BANPs groups where rats were orally gavaged PBS, *B. amyloliquefaciens and B. amyloliquefaciens* loaded nanoparticles (BA at level of 1.0 × 10^10^ CFU/kg in 1 mL of PBS/rat/day), respectively, for 7 days. Colitic groups: DSS, BA and BANPs groups where rats were orally gavaged dextrane sodium sulphate (DSS), *B. amyloliquefaciens* + *DSS and B. amyloliquefaciens* loaded nanoparticles (BA at level of 1.0 × 10^10^ CFU/kg in 1 mL of PBS/rat/day) + *DSS*, respectively, for 7 days. All groups orally gavaged by 5% DSS. Values are expressed as mean ± SE, ^a,b,c^Means of the bars with different letters were significantly different among groups (*P* < 0.05).

The levels of MDA, NO and TAC are illustrated in Fig. 4a–c, respectively. Remarkably, NO and MDA levels were significantly increased (P < 0.05), (increased by 360% and 230% vs non-colitic group) while TAC was significantly reduced in DSS induced group). Meanwhile, administration of BANPs to DSS induced group significantly decreased (P < 0.05) NO and MDA levels when compared with DSS induced group (decreased by 242% and 196% vs colitic group). Notably, DSS induction diminished the levels of TAC, in contrast administration of free BA or BANPs significantly increased (P < 0.05) TAC regardless to DSS induction.

### Quantification of inflammatory makers in colon by ELISA and real-time PCR

**Figure 5:**
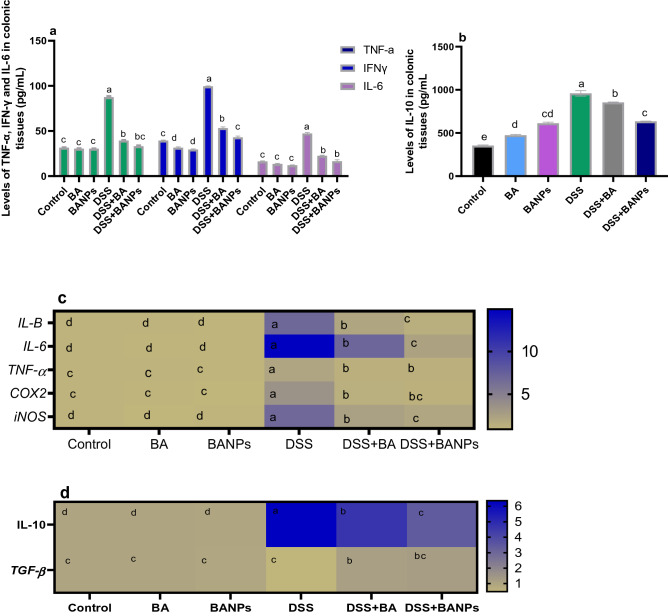
Effects of orally administered *B. amyloliquefaciens (BA) or BA-nanoparticles (BANPs)* in non-colitic and colitic rats on measurement of cytokines protein levels by ELISA kits (**a**,**b**) and heat map illustrating mRNA of various inflammatory markers including cytokines by real time PCR (**c**,**d**) 7 days post DSS induction. Interleukin (IL)-1β, IL-6, IL-10, tumor necrosis factor α (TNFα), transforming growth factor-beta (TGF-β), cyclooxygenase-2 (COX-2) and Inducible nitric oxide synthase (iNOS). Non-colitic groups including Control, BA and BANPs groups where rats were orally gavaged PBS, *B. amyloliquefaciens and B. amyloliquefaciens* loaded nanoparticles (BA at level of 1.0 × 10^10^ CFU/kg in 1 mL of PBS/rat/day), respectively, for 7 days. Colitic groups: DSS, BA and BANPs groups where rats were orally gavaged dextrane sodium sulphate (DSS), *B. amyloliquefaciens* + *DSS and B. amyloliquefaciens* loaded nanoparticles (BA at level of 1.0 × 10^10^ CFU/kg in 1 mL of PBS/rat/day) + *DSS*, respectively, for 7 days. All groups orally gavaged by 5% DSS. Values are expressed as mean ± SE, ^a,b,c^Means of the bars with different letters were significantly different among groups (*P* < 0.05). The scale bar on the right side described the unit for each color as blue and light green squares corresponded to significant upregulation and downregulation, respectively of genes relative to the control groups (P < 0.05).

Quantification of cytokines by ELISA kits are presented in Fig. 5a,b. DSS induced group showed the highest inflammatory response as detected by highest levels (P < 0.05) of proinflammatory cytokines (IL-1β and TNF-α). Meanwhile their levels were reduced (P < 0.05) in group received BANPs followed by group received free BA. Among all DSS induced groups, the level of IL-6 only reduced in group received BA NPs (decreased by 65% compared to DSS induced group). In non-colitic group, BANPs or BA received groups had a significant higher (P < 0.05) levels of IL-10 compared to control. In contrast, DSS induced group displayed higher levels of IL-10 (P < 0.05) than DSS induced groups treated with BA or BANPs. In accordance with ELISA detection, mRNA expression for the pro-inflammatory cytokines, *TNF-α, IL-1β* and *IL-6*, and the anti-inflammatory cytokine *IL-10* were highly expressed in DSS group compared with that in the BANPs treated group (Fig. 5c,d). Notably, colonic inflammation in DSS indued group reduced the expression of *TGF-β*, unlike group received BANPs exhibited higher *TGF-β* expression (increased by 0.75 fold compared to DSS control group). Interestingly, BA or BANPs decreased the mRNA of *COX-2* and *iNOS*, an inflammatory marker, (Fig. 5c) compared DSS induced group (decreased to 1.32- and 1.13-fold vs 3.5 and 2.5 and 1.9 vs 6.8, respectively).

### Expression of apoptotic targeted genes

**Figure 6:**
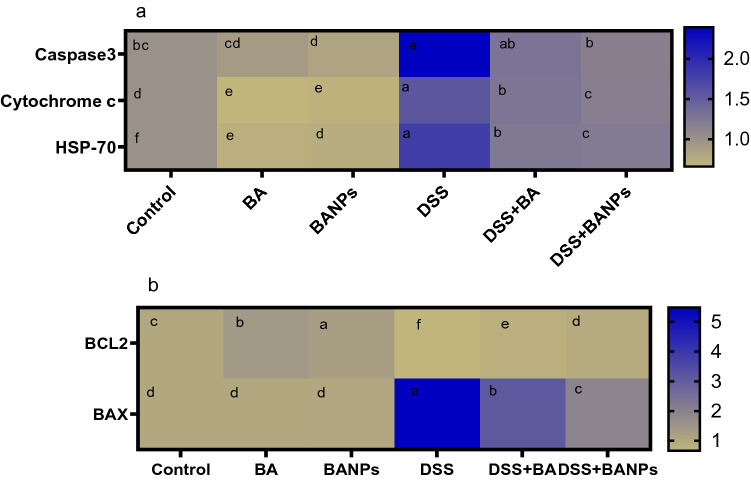
Heat map illustrating the effects of orally administered *B. amyloliquefaciens (BA) or BA-nanoparticles (BANPs)* in non-colitic and colitic rats on mRNA expression of caspase-3, heat shock protein-70 (HSP-70), Cytochrome c, Bax and Bcl-2, 7 days post DSS induction. Non-colitic groups including Control, BA and BANPs groups where rats were orally gavaged PBS, *B. amyloliquefaciens and B. amyloliquefaciens* loaded nanoparticles (BA at level of 1.0 × 10^10^ CFU/kg in 1 mL of PBS/rat/day), respectively, for 7 days. Colitic groups: DSS, BA and BANPs groups where rats were orally gavaged dextrane sodium sulphate (DSS), *B. amyloliquefaciens* + *DSS and B. amyloliquefaciens* loaded nanoparticles (BA at level of 1.0 × 10^10^ CFU/kg in 1 mL of PBS/rat/day) + *DSS*, respectively, for 7 days. All groups orally gavaged by 5% DSS. Values are expressed as mean ± SE, ^a,b,c^Means of the bars with different letters were significantly different among groups (*P* < 0.05). The scale bar on the right side described the unit for each color as blue and light green squares corresponded to significant upregulation and downregulation of genes relative to the control groups (P < 0.05).

As illustrated in (Fig. 6). BANPs attenuated apoptosis of inflamed colon as revealed by a 2.94fold decrease of caspase-3 mRNA expression. In the same context, decreased mRNA expression of anti-apoptotic (Bcl-2) and increased pro-apoptotic genes as cytochrome c and Bax was observed in DSS control group. Meanwhile, the expression of Bcl-2 was increased by 0.3 fold and cytochrome c and Bax were decreased by 0.2 and 3.55 fold in group treated with BA NPs, respectively when compared with DSS control group.

### Microbial populations of colon contents

**Figure 7:**
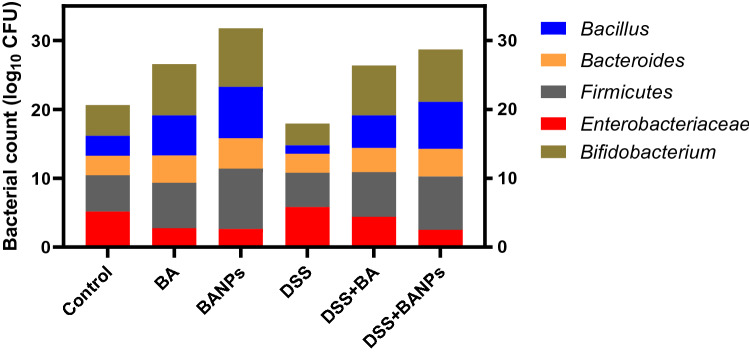
Effects of orally administered *B. amyloliquefaciens (BA) or BA-nanoparticles (BANPs)* in non-colitic and colitic rats on abundance of *Bacillus*, *Bacteroides*, *Firmicutes*, *Enterobacteriaceae* and *Bifidobacterium* populations (log_10_ CFU), 7 days post DSS induction. Non-colitic groups including Control, BA and BANPs groups where rats were orally gavaged PBS, *B. amyloliquefaciens and B. amyloliquefaciens* loaded nanoparticles (BA at level of 1.0 × 10^10^ CFU/kg in 1 mL of PBS/rat/day), respectively, for 7 days. Colitic groups: DSS, BA and BANPs groups where rats were orally gavaged dextrane sodium sulphate (DSS), *B. amyloliquefaciens* + *DSS and B. amyloliquefaciens* loaded nanoparticles (BA at level of 1.0 × 10^10^ CFU/kg in 1 mL of PBS/rat/day) + *DSS*, respectively, for 7 days. All groups orally gavaged by 5% DSS.

The effects of orally administered *BA or BANPs* in non-colitic and colitic rats on abundance of colon bacterial populations are shown in (Fig. 7). Feeding on *BA or* BANPs significantly increased the abundance of beneficial bacterial population and unlike decreased the counts of pathogenic ones. More detailly, compared with the DSS group, colitic rats received *BANPs* displayed the higher *Firmicutes* counts (4.97 vs 7.73, respectively). Additionally, the numbers of beneficial bacterial population *(Lactobacillus* and *Bifidobacterium)* were elevated in colitic groups received BANPs (increased by 5.6 and 3.53 CFU/g of the colon contents) then BA (increased by 4.46 and 4.1 CFU/g of the colon contents) compared to DSS induced group. Remarkably, both non-colitic and colitic group received BANPs showed the lowest population of *Enterobacteriaceae in colon contents*.

### Histological changes in the colon

**Figure 8:**
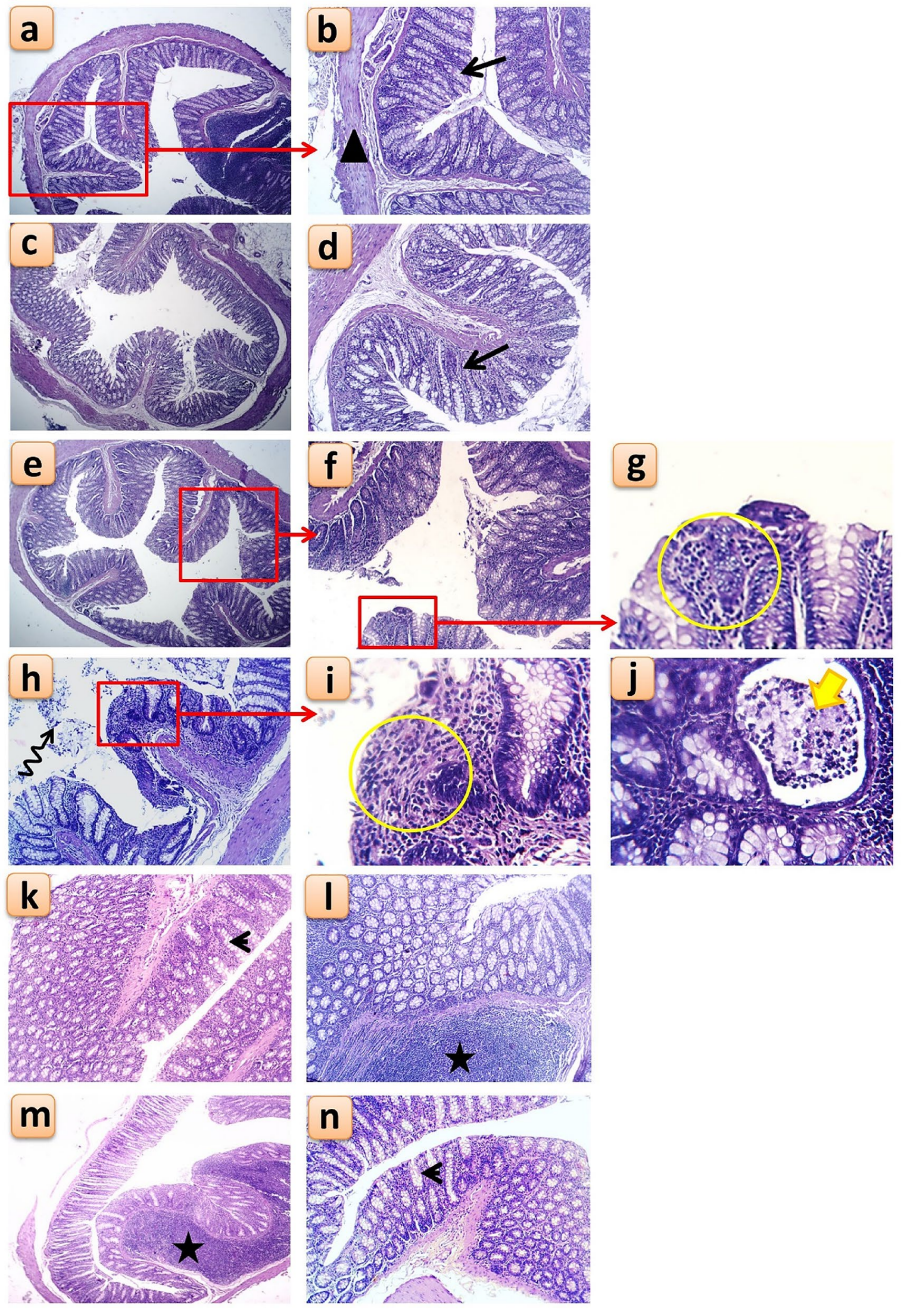
Histological changes following oral administration of *B. amyloliquefaciens (BA) or BA-nanoparticles (BANPs)* in non-colitic and colitic rats. Non-colitic groups including Control (**a**,**b**), BA (**c**,**d**) and BANPs (**e**–**g**) groups. While, colitic groups include DSS (**h**,**j**), DSS + BA (**k**,**l**) and DSS + BANPs (**m**,**n**) groups*.* Red rectangle = magnification of selected area, Arrow = normal colon crypt structure, black triangle = normal muscularis mucosa layer, yellow circle = diffuse leukocytic infiltration, zigzag arrow = desquamated colon epithelium, thick yellow arrow = mucosal abscess within the colonic crypts, arrow head = apparently normal colon crypts with increased number of goblet cells, star = prominent lymphoid aggregation within the submucosa.

In the non-colitic control groups, Control, BA and BANPs groups, the histological examination showed normal histological layers of the colon which include; mucosal columnar epithelium with goblet cells, sub mucosal layer and muscular coat (Fig. 8a,b). In the BA group, there was a slightly improved colonic mucosa represented by focal areas of regenerative attempts of colon crypts (Fig. 8c,d), while the BANPs group showed reconstructed colon epithelium in some areas with fewer inflammatory cells infiltration and reduced detachment of epithelial lining (Fig. 8e–g).

This picture was completely changed in the colitic DSS (positive control) group which revealed diffuse colitis manifested by necrotic epithelial lining mucosa with depleted goblet cells, mononuclear leukocytic infiltration within mucosa and surrounding colonic glands in submucosa (Fig. 8h). In addition to some intestinal glands were damaged surrounded by severe leukocytic infiltration and others were cystically dilated (Fig. 8i). Moreover, desquamated cells within colon lumen (Fig. 8h) and small mucosal abscess were also seen (Fig. 8j). While, following administration of BA, in the DSS + BA the colitic histological picture was much reduced with less necrotic epithelium of the mucosa, less depleted goblet cells with increased secretory activity and reduced mononuclear leukocytic infiltration, however, prominent lymphoid aggregations were still evident (Fig. 8k,l). Whereas, in the DSS + BANPs group, receiving BA-loaded nanoparticles, showed the best improvement within all colitic groups, with preserved colonic histological architecture, hyperactivity of submucosal glands, fewer submucosal lymphoid nodules and mucosal crypts with abundant goblet cells (Fig. 8m,n).

### Immunohistochemical detection of Ki67 and IL-6 in the colon

**Figure 9:**
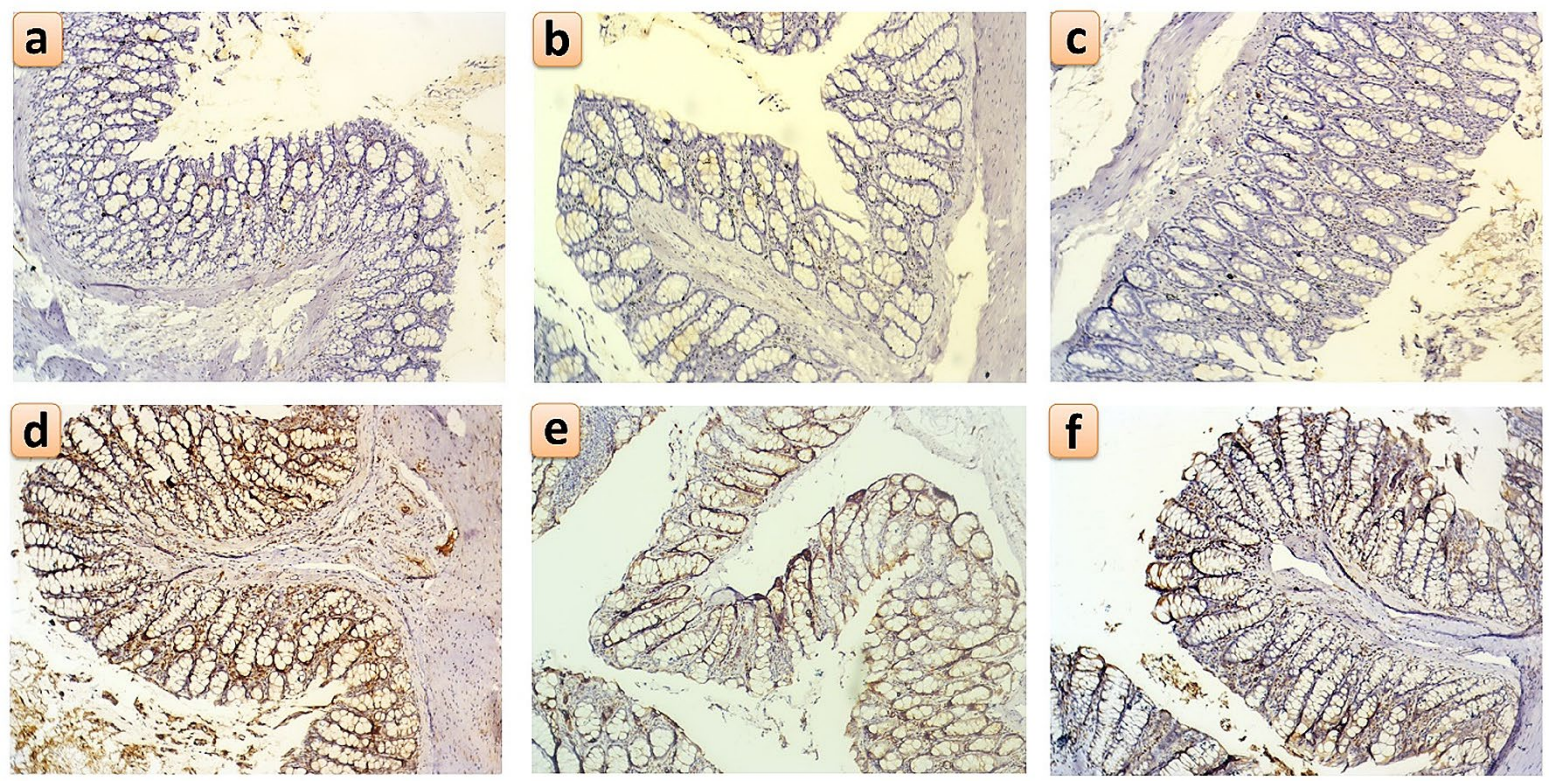
Immunohistochemical staining for IL-6 positive cells within the colonic mucosa and submucosa following oral administration of *B. amyloliquefaciens (BA) or BA-nanoparticles (BANPs)* in non-colitic and colitic rats. Non-colitic groups including Control (**a**), BA (**b**) and BANPs (**c**) groups. While, colitic groups include DSS (**d**), DSS + BA (**e**) and DSS + BANPs (**f**) groups*.*

**Figure 10:**
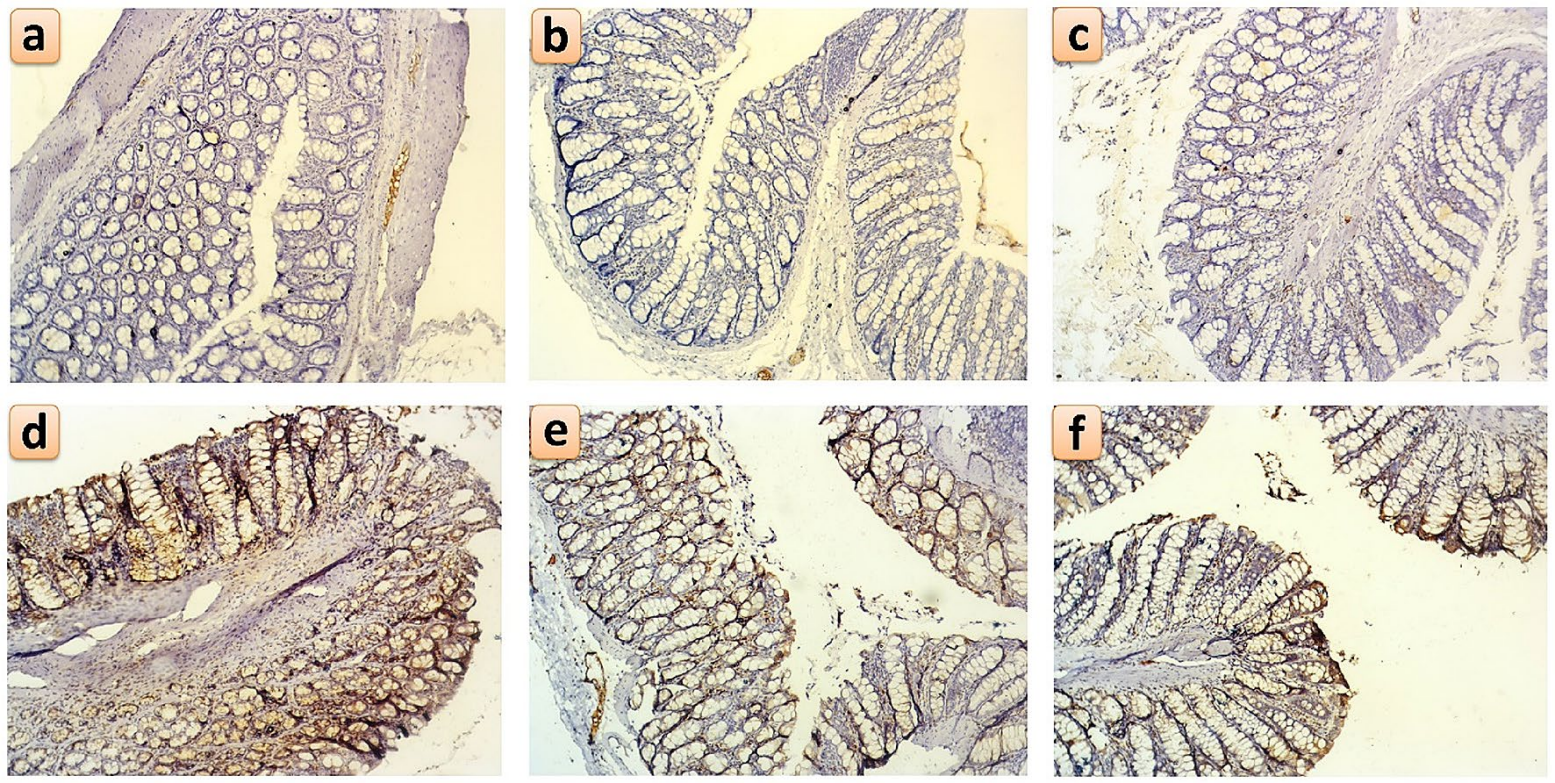
Immunohistochemical staining for Ki-67 positive cells within the colonic mucosa and submucosa following oral administration of *B. amyloliquefaciens (BA) or BA-nanoparticles (BANPs)* in non-colitic and colitic rats. Non-colitic groups including Control (**a**), BA (**b**) and BANPs (**c**) groups. While, colitic groups include DSS (d) , DSS + BA (**e**) and DSS + BANPs (**f**) groups*.*

In the non-colitic groups, Control, BA and BANPs groups, there were sporadic IL-6 expressing cells in colonic mucosa in the lamina propria between the glandular crypts and scattered in the submucosa (Fig. 9a–c, respectively). While in the DSS (positive control) group, the highest expression of IL-6 was diffusely seen in both the glandular epithelium as well as the submucosa (Fig. 9d). In the DSS + BA and DSS + BANPs groups, IL-6 immuno-positive cells’ number were greatly reduced following administration of BA or BANPs (Fig. 9e,f, respectively). In addition, Ki-67 expression profile followed a similar pattern as IL-6. In the non-colitic groups, Control, BA and BANPs groups, no or scanty Ki-67 expressing cells were found in the lamina propria of the mucosa and in the submucosa (Fig. 10a–c, respectively). Following induction of IBD by DSS, there was a strong expression of Ki-67 throughout the colonic mucosa and submucosa (Fig. 10d), while after administration of BA or BANPs, there was a significant reduction in Ki-67 expressing cells in the colonic sections, and especially in the lamina propria and the submucosa (Fig. 10e,f, respectively).

## Discussion

The current conventional therapeutics used for treatment of IBD are mainly anti-inflammatory agents, consisting of corticosteroids and 5-amino salicylic acid and its derivatives as sulfasalazine^20^. However, application of these drugs are restricted by their non-specific actions on immune system that resulted in short- and long-term debilitating side effects, such as nausea, allergic reactions, pancreatitis, elevated liver tests, and other life-threatening side effects^21^. Thus, there is an urgent need for searching inexpensive an alternative agent that may be equally or more effective than used conventional drugs. Targeting natural ingredients based on nanoparticles have been designed and displayed a great potential for improving IBD treatment. While screening the beneficial impacts of probiotics strains, *Bacillus amyloliquefaciens (BA)* is recognized to be a promising safer alternate to antibiotics with no side effects^22^. This directed our attention towards improving properties of probiotics in treating colitis by their incorporation in a nano formula. To the best of our knowledge, there are little reports available in the literature concerning the efficacy of oral administration of probiotic-loaded nanoparticles (*Bacillus amyloliquefaciens* loaded chitosan and sodium alginate nanoparticles (BA loaded NPs) on treatment of colitis. In the previous study, administration of *Bacillus amyloliquefaciens* had beneficial effects on DSS-induced colitis, proposing that this strategy might be effective for the treatment of colitis^23^. Additionally, current study demonstrated that development of novel nano-delivery system for BA enhanced its stability and mode of action along the gastrointestinal tract thus being more effective for treating IBD than free BA. In accordance, application of novel nano-vehicle for delivery of probiotics strains increased their survival and stability along gastrointestinal tract (GIT)^17^. Additionally, entrapping of probiotics in to a nano system has many advantages as to maintain probiotics stability, deliver a barrier to protect them from damage, to isolate bacteria from their environmental conditions, to provide a carrier with a high probiotics load, to allow controlled and continuous probiotics release, to enable probiotic adherence and prolong residence time^24,25^. All these functions augmented their oral colon-targeted therapy. Our resulted concerning survival of BANPs in activated gastric and intestinal juice support their stability more than free BA that in agreement with^26^.

Remarkably, our study demonstrated that oral administration of BANPs significantly ameliorated loss of body weight, reduced spleen enlargement in DSS-induced rats. Unlike, DSS induced and non-treated rats lost up to 20% of their body weight. Similarly, DSS induced mice exhibited a pronounced weight loss (at day 5, approximately 5–10% reduction) and altered stool consistency with consequence of diarrhea and hematochezia^27^. On the other hand, spleen enlargement is a proving organ index of inflammation and there are relationship between colitis and enlargement of spleen size in animals^28^. Notably, the colon length and spleen weight of BANPs treated group were nearly similar to control non DSS-induced rats. It was stated that colon length is inversely correlated to the severity of DSS-induced colitis^29^. These beneficial impacts following BANPs administration indicated a superior ameliorative effect of BA loaded NPs on treating colitis. In accordance, nanoparticles coating of *Lactobacillus acidophilus* and *Lactobacillus rhamnosus* protect them from degradation in the gastrointestinal region (low pH in the stomach and bile salt in the small intestine) thus augmented their viability and support their mode of actions^30^. Effective therapeutic impact of BANPs in colitic rats may be attributed to its nano delivery system that allow great promise protection for microorganisms from stomach acidic conditions and therefore allowing successful probiotics release in the intestinal lumen with natural pH that in accordance with^31^.

Fecal Lcn-2 widely accepted as biomarker for intestinal inflammation^32^. Herein, Fecal Lcn-2 levels were reduced at day 4 of DSS induction in BANPs received group. In contrast, DSS induced and non-treated rats displayed higher levels of Lcn-2 over 7 days. Reducing the inflammatory markers (Lcn-2 levels) in DSS induced rats following treatment with BANPs remarkably revealed recovery from inflammation in this group unlike DSS induced and non-treated group which in consistent with^33^. DSS induced colitic animals showed developed bloody diarrhea, a significant weight loss, and anemia manifested by decreased levels of RBC and Hb^34^. Unlike, in the current study, administration of BANPs after DSS induction restored levels of RBC and Hb. Herein, liver and kidney functions tests were distributed in DSS induced rate, while administration of BA probiotics or BA NPs were capable of restoring their functions, by stopping the leakage of hepatic enzymes into circulation, maintaining the integrity of hepatocyte membrane and significantly providing hepatoprotective effect. Similarly, DSS induced rats showed a significant alteration of hepatic and kidney functions by increase of these parameter^35^.

Increase lipid peroxidation of DSS may indicate a potential mechanism of tissue injury by reactive oxygen species^36^. MPO activity, a good marker of tissue inflammation, is among most important factors that reflect infiltration of granulocyte into colonic tissues^37^. Its activity in the colon is linearly related to infiltration of neutrophil^38^. Additionally, neutrophils infiltration triggers excessive production of reactive oxygen species (ROS), and nitric oxide (NO) which ultimately provoke intestinal injury^39^. Interestingly, treatment of DSS induced rats with BANPs reduced serum NO levels, MPO activities compared with DSS induced and non-treated rats. These data indicated that BANPs attenuated leukocyte invasion and mucosal damage in DSS-induced colitis. In the current study, the reduced levels of MPO in BANPs administrated rats, when compared to those in DSS-induced rats, suggested the beneficial effects of alleviating inflammatory responses produced by the granulocytes. Serum CRP is a biomarker of colitis and its levels was correlated with severity of colitis^40^. Herein, induction of colitis by DSS evoked an elevated CRP levels in serum when compared to the control non induced group. Meanwhile, BANPs treatment reduced CRP levels in the serum reflecting their positive therapeutic effect on colitis. These positive findings can be attributed to incorporation of BA into polymeric nanoparticles that allow its delivery to the desired site^41^ and can be used to conquer cellular barriers^42^.

Along the course of colitis, the inflammatory process elicits oxidative stress and reduces cellular antioxidant capacity^43^. In the present study, induction of colitis caused a marked oxidative stress as reflected by higher of MDA and NO levels along with reduced TAC levels unlike colitic group. In contrast, BANPs afforded significant protection against oxidative stress and boosted the antioxidant status in rats with DSS as evidenced by decreased levels of MDA and NO and reinstatement the activities TAC, as compared to DSS colitis group. These observations are in line with previous studies^44–46^. Similarly, *Bacillus* probiotic strains, have been shown to display strong antioxidant capacity and attenuate oxidative damages both in vitro and in vivo^47,48^. The possible antioxidant effects mechanisms of probiotics comprising, self-secretion of antioxidant metabolites and reducing enzyme activities that mediate ROS production^49^. All these explanation could augment the role BANPs in decreasing the risk inflammation after colitis induction and in agreement with^50^.

Next to the colitis induction by DSS a variety of tissue derived cytokines have been shown to be increased as early as the first day of DSS-induction^51^. Pro-inflammatory cytokines are critical biomarkers which are released in intestinal mucosa with immune cells hyper-activation following IBD induction. After immune cells stimulation, there is an elevation in cytokines levels (TNF-α, IL-1β and IFN-γ). TNF-α, IL-1β, and IL-6 are released in response to tissue injuries and infections. Moreover, dysregulated and persistent of IL-6 synthesis has a pathological influence on autoimmunity and colonic inflammation, however expression and secretion of IL-10 can protect from colitis^52^. Additionally, the overproduction of such inflammatory mediators is strongly linked to IBD pathogenesis. As, this elevated inflammatory reaction produces additional ROS, triggering cytokines and proteolytic enzymes which resulted in tissue damage^53^. Herein, serum and transcriptional levels of pro-inflammatory cytokines (including IL-6, IL-1β, and TNF-α) were reduced and anti-inflammatory cytokines (IL-10) were increased in group administrated BANPs suggesting beneficial role of administrated BANPs in augmenting colonic epithelial amelioration. Meanwhile, data concerning colitic non treated group showed an excessive increased level of pro-inflammatory and anti-inflammatory cytokine. Heretofore, upregulation of IL-10 has been stated in colons of rats with TNBS- and dextran sulfate-induced colitis^54^. Higher circulating IL-10 levels can be noticed as a compensatory mechanism against colonic damage and is suggested to play a key role in restricting mucosal inflammation through reducing the presentation of MHC class II antigen and consequent pro-inflammatory cytokines release. However, the increased IL-10 levels in the current might not be sufficient to completely control colon inflammation that in agreement with^55^. In accordance, BA supplementation decreased the levels of pro-inflammatory cytokines in DSS-treated mice^14^ which could clarify the superior function of BANPs treatment. Herein, the noted IL-10 restoration by BANPs probably suggested an improvement of the inflammatory response which was related to proinflammatory signals inhibition. In this context, the detected BANPs lowering effect of TNF-α may be involved in the alleviation of colonic IL-10 levels, while IL-10 release is affected by higher proinflammatory cytokines levels^54^. Therefore, the current results reinforce the relieving actions of BANPs in DSS colitis resulted from to its anti-inflammatory actions. In agreement, *Bifidobacterium bifidum* supplementation significantly elevated of IL-10 levels and decreased the of IL-6 levels in the colon sections, which proved the anti-inflammatory action^56^. These findings are in agreement with preceding researches demonstrated that addition, *Lactobacillus acidophilus* maintaining remission in colitis^57^. Previously, oral administration of probiotics exhibited significant potential as an alternative treatment of IBD due to their capacity to reestablish the homeostasis of gut microbiota, restore the integrity of gut barrier, modify immune status, protect against invading pathogens and hinder chronic inflammation^58^. Moreover, research has showed that alginate and chitosan have mucoadhesive properties and can adhere to the mucus to expand residence time, therefore accelerating probiotics colonization in the intestine^59^. The mechanisms implicated in the optimistic effects of probiotics in the course of IBD comprise reduction of pathogenic microorganism, modulation of immune system and production of substances concerned in proliferation and maturation of cells, such as short-chain fatty acids^21–23,60^. In this line of thought, *B. longum* ATCC 15707 is capable to beneficially modify the immune system by IL-10 release and could consequently be used in managing IBD^61^. Herein, superiority of BANPs in facilitating colonic epithelial amelioration could be comprehended by improving its survival and resistance in gastro-intestinal conditions; therefore, increased the lifetime of cells.

Excessive cytokines generation and ROS has been described to activate various transcription factors that stimulate the inflammatory response. Among them, the nuclear factor kappa B (NFkB) triggers of proinflammatory genes transcription involving *COX*-2 and *iNOS*^43^. During course of colitis, upregulation of COX-2 produces an arsenal of PGE2 and TXB2 which aggravates hyperemia and edema whereas in the intestine, while activation of iNOS led to NO release which weakens the integrity of colon by peroxynitrite synthesis, a powerful oxidizing agent which is formed through superoxide anion and NO reaction^62^. In contrast, their expression was restored in DSS induced group after treatment with BA loaded NPs. In agreement, beneficial *Lactobacillus* reduced the production COX-2, nitric oxide synthase NOS and pro-inflammatory cytokines such as TNF-α and IL-6^63,64^. Similarly, ^65^proved that the oral administration of *L. plantarum* strain (CAU1055) significantly reduced the expression levels of *TNF-α, IL-6, iNOS,* and *COX-2*. The observed downstream of inflammatory effectors as *COX-2* and *iNOS* is considered as an advantage of BANPs in colitis management and can be attributed to releasing of their metabolic factors^66^. We further found that BANPs altered mitochondrial proteins expression, such as Hsp70 (are synthesized in response to injury^67^. Heat shock response is triggered in active colitis and can induce proinflammatory cytokines activation involved in inflammatory bowel disease pathogenesis^68^. In the same line, treatment with active metabolites of beneficial gut microbiota as butyrate resulted in inhibition of increased expression of HSP70 thus provide protection against mucosal injury as in case of colitis^69^. Herein, the enhanced effect of BANPs than free BA on attenuation of induced colitis can be attributed to their incorporation in nano form that plays an important role in their survival and stability thus augmenting their mode of action in GIT^17^.

Our data also expressed an apoptosis activation in colonic tissues as shown by upregulation cytochrome c, and caspase-3 pro-apoptotic genes as well as downregulation of the anti-apoptotic genes expression of *Bcl-2* and *Bax* was noticed in DSS induced rat which in accordance with finding in with^70,71^. Higher rate of colonic apoptosis has been stated in TNBS induced colitis and patients suffered from ulcerative colitis and^71^. It has been described that the oxidative stress prompts numerous genes expression accountable for death of cells via apoptosis^71^. Bcl-2 family including Bcl-2 and Bax has an important role in controlling apoptosis. Bcl-2 is considered as a pro-survival signal and Bax is a pro-apoptotic member as it binds and antagonizes of Bcl-2 actions^72^. Higher ratio of Bax/Bcl-2 can stimulate cytochrome c release from mitochondria to cytosol, which initiates caspase-9 and eventually *caspase*-3^71^. Our data showed that BANPs upregulated *Bcl-2* with downregulation of anti-apoptotic *Bax* expression and the pro-apoptotic *cytochrome c*, and *caspase-3* expression, demonstrating their role in colonic apoptosis attenuation. These findings are agreement with previous studies that mitigation of colonic apoptosis can be related to inhibition of oxidative stress as increased exposure of intestinal mucosa under inflammatory stimuli to ROS augments epithelial apoptosis^73^.

Additionally, novel therapies for treating bowel inflammatory diseases based on modifying gut microbiota, have been received much attention^74^. Herein, administration of BA had a positive impact on modulation of colon microbiota toward beneficial ones. Moreover, their incorporation into nano particles clearly augmented this function. As increasing of *Lactobacillus* and *Bifidobacterium* had an important role in reducing oxidative damage specially after induction of colitis which in accordance with^75^. Bacteroides able to degrade organic matter with high-molecular weight and improve the innate immune responses by boosting intestinal barrier function^76^. Additionally, *firmicutes* have many cellulolytic organisms, which are helpful for decomposition of cellulose^77^. Moreover, *Bacteroidetes* to *Firmicutes* ratio is a reliable index to evaluate the gut microbial composition^78^. Colitic group received BANPs had a prominent shift to an increased *Firmicutes* to *Bacteroidetes* ratio that was considered to be accountable for protection against inflammatory bowel disease^23,79^. These positive effects resulted from Incorporation of BA into a nano particle that enhanced its survivability in gastrointestinal tract consequently modifying microbial communities.

Following induction of IBD using DSS, the colon histological examination showed a complete destruction of colon epithelial architecture with diffuse inflammatory cells infiltration and loss of the colon crypts. These typical histological changes were mucin and goblet cell depletion, epithelial erosion/ulceration and infiltration of leukocytes into the lamina propria and submucosa^80^, indicating the severe activation of the local immune responses. These macroscopic and microscopic alterations that we observed in this study agree with numerous reports of DSS-induced colitis as a model of IBD in rats^81^. While, after the administration of BA or BA-loaded nanoparticles in colitic rats these histological changes were greatly reduced and the histoarchitecture of the colon was restored to near normal condition, with healthier colonic mucosa, crypts, mucosal glands and submucosa was less infested with inflammatory cells, these effects were more in the group receiving BA-loaded nanoparticles than the group receiving BA alone.

In addition, Ki-67 is considered an important marker for the evaluation of dysplastic changes in colon mucosa in colitis^82,83^. In the present study, the expression of Ki-67 was greatly affected by the treatments, where the highest levels of Ki-67 positive cells were found dispersed in the whole colon section (both in the mucosa and submucosa) in the DSS group. These Ki-67 positive cells were reduced following administration of BA or BANPs, but the best reduction was found in the group receiving BANPs. Similarly, IL-6 positive cells were increased following DSS induction of colitis, these cells were also dispersed throughout the mucosa and submucosa and their levels were greatly reduced after treatment with BA or BANPs, and the best reduction was also achieved in the BANPs receiving group. Previous reports showed that IL-6 is one of the main inflammatory contributors to the various pathological consequence following colitis^52^. It was also shown to be strongly related to the progression towards malignancy and the development of colitis associated cancer^84,85^. Therefore, BA-loaded nanoparticles administration can lead to less tendency to the development of severe colonic inflammation and, therefore, reduced predisposition to the occurrence of colon malignancy. Moreover, these visible histological and immunohistochemical enhancements observed in the BA-loaded nanoparticles group substantiate the biochemical, oxidative stress and gene expression findings reported in this study and highlight the promising therapeutic potential of BA-loaded nanoparticles in reducing the severity of IBD and reducing its associated inflammatory response^86^.

## Conclusions

This study signifies for the first time that a novel, natural, nano delivery system of beneficial probiotics strains is capable of targeting the inflamed intestinal mucosa and stopping damaging factors while promoting healing factors. Herein, treated rats with BANPs showed a reduced intestinal damage following DSS induced colitis as well as an improved recovery from colitis than used free BA. Thus, these findings promote the use of BANPs as an effective therapeutic strategy for alleviating colitis.

## Methods

### Synthesis and characterization of probiotics nano particles

**Figure 11:**
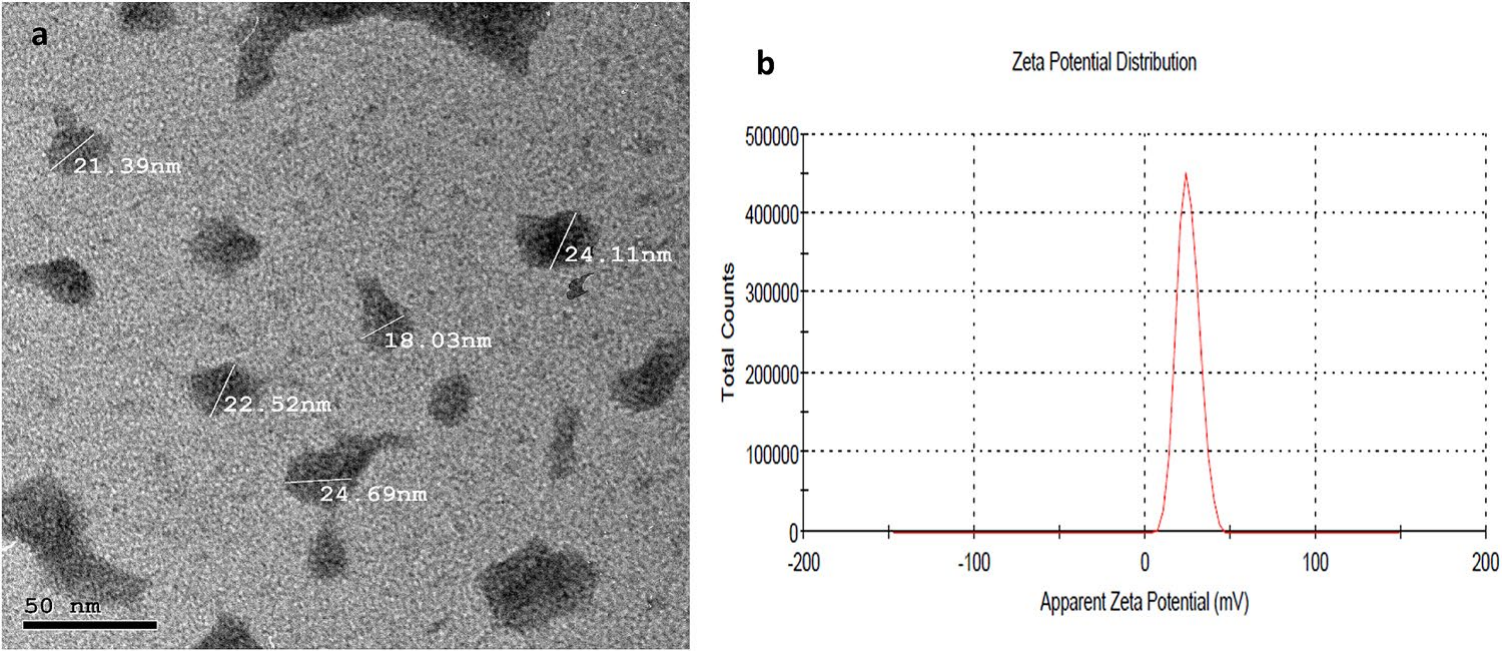
Transmission electron microscopy (**A**) and zeta potential distribution (**B**) of *B. amyloliquefaciens* loaded nanoparticles.

Chitosan (Sigma-Aldrich) and probiotic organisms (*Bacillus amyloliquefaciens*) and were incorporated into of sodium alginate (10 mL of 20 g/L, Sigma-Aldrich). Aqueous solution of chitosan was prepared. Briefly, chitosan was dissolved in distilled water (100 mL) acidified with glacial acetic acid to attain a final concentration of chitosan (0.4%, w/v). In the next step, previous solution was filtered via a nylon cloth for removing of any residual insoluble material. The sodium alginate solution was extruded into a former sterile chitosan solution and stirred forming beads, then these beads were sieved off from chitosan solution and washed with sterile distilled water. The capsulates were held in cryoprotectant agent and then frozen at − 20 °C. The frozen samples were dried under vacuum, at a condenser for about 18 h at − 40 °C with a freeze-drier (Freezedryer Lyobeta25). Dried cells were stored under darkness at 4 °C. The characterization of prepared probiotics nanoparticles was done by transmission electron microscopy (TEM) and zeta potential distribution Fig. 11.

### Survival of *Bacillus amyloliquefaciens* loaded nano particles cells after incubation in simulated gastric juice and simulated intestinal juice

This analysis was based on the method described by^18^. Freshly prepared coated coated beads (1 × 10^10^ log cfu/g bead) and 1 mL of free bacteria suspension were placed in a tube with 10 mL sterile activated gastric juice (0.08 m HCl including 0.2 g/100 mL NaCl, pH 1.55) without pepsin and incubated at 37 °C for 30, 60, 90, and 120 min. After incubation, the beads and free bacterial suspension were harvested and placed in 9 mL of sterile activated intestinal juice (0.05 mol/L of KH2PO4, pH 7.5 with or 0.6% bile salt). After that the tubes were incubated at 37 °C for 150 min. After incubation, a 1 mL aliquot containing dissolved free bacterial cells and beads of each bacterium was removed form activated intestinal juice and enumerated as cell count (CFU/mL) on tryptic soy broth (TSB, Becton Dickinson, Sparks, MD, USA) at 37 °C for 24 h.

### Experimental animals and DSS-induced colitis model

All animal experiments were performed in compliance with the guidelines and regulations approved by the Institutional Animal Care and Use Committee (ZU-IACUC/2021), Faculty of Veterinary Medicine, Zagazig University. All animal protocols were carried out in compliance with the ARRIVE guidelines. Adult Wistar rats (male, aged 6–7 weeks, 200 ± 20 g) were used and 5% (w/v) DSS was used to induce acute colitis mice model. Animals were maintained at controlled environmental conditions at temperature (22 ± 1 °C), humidity (50 ± 5%) and acclimated for one week before any experimental procedures. Based on the weight, all rat were randomly distributed into 6 groups as follows: three non-colitic groups; rats were received standard diets and fresh drinking water for 7 days then orally gavaged for 7 days by PBS, (Control group), *B. amyloliquefaciens* (BA at level of 1.0 × 10^10^ CFU/kg in 1 mL of PBS/rat/day) or BA loaded NPs (BA-NPs 1.0 × 10^10^ CFU/kg in 200 μL of PBS/rat/day). The remaining three colitic groups sets as: rats were supplemented with 5% DSS (w/v; molecular mass = M_w_ 7000–20,000; product No. 51227-100G, Sigma-Aldrich, Shanghai, China) dissolved in fresh running water ad libitum for 7 days and then orally gavaged for other 7 days by PBS, (IBD control group), *B. amyloliquefaciens* (BA at level of 1.0 × 10^10^ CFU/kg in 1 mL of PBS/rat/day) or BANPs (BANPs 1.0 × 10^10^ CFU/kg in 200 μL of PBS/rat/day). The probiotic *B. amyloliquefaciens* was CECT 5940 and provided by Evonik Operations GmbH).

### Assessment of colitis clinical signs and severity

Throughout the experimental period, body weight gain, and physical activity were monitored daily. Clinical signs were observed daily, and stool were examined for the presence of blood or water consistency to assess the severity of colitis. Colonic length, spleen weight, stool consistency, rectal bleeding and were evaluated and scored as described by standard protocols^87^.

### Sampling

At the end of the experiment, rats were anesthetized intravenous by ketamine hydrochloride, (30 mg/kg BW) then euthanized by cervical dislocation. Blood was collected for hematological finding in heparinized tubes and for serum collection (centrifuged at 4 °C at 4000 rpm for 10 min). Fecal samples were collected and used for evaluation of fecal Lipocalin-2 marker. The colon tissues (the distal 10 cm portion of the colon) were taken, cleaned with PBS to remove any faecal residues and cut into two portions. One section was placed on filter paper, pinned at the ends, fixed in 4% paraformaldehyde for hematoxylin and eosin (HE) staining, and assessed under a light microscope. The other colon segment was stored directly at − 80 °C for further analysis of genes expression.

### Measured parameters

#### Disease activity index (DAI)

The scores of DAI ranged from 0 (healthy) to 12 (severe colitis) were measured as previously according to^88^. The overall disease severity was evaluated with a clinical scoring system as the % body weight loss (score 0–4), rectal bleeding (scores 0–4), and stool consistency (score 0–4) were evaluated (Table 3). These values were considered for each animal, and the sum of the 3 values represented the DAI. Diarrhea was evidenced by incidence of mucus on rat feces. Meanwhile, fecal matter was used for rectal bleeding assessment (ranged from occult blood to gross bleeding).

**Table 3:** Scoring of disease activity index (DAI).

SCORE	Weight loss	Stool consistency	Rectal bleeding
0	None	0 = normal	0 = negative
1	0.1–5%		
2	5–10%	2 = loose stool	2 = Hemoccult
3	10–20%		
4	4 = 20%	4 = diarrhea	4 = gross bleeding

#### Hematological and serum analysis

Red blood cell counts (RBC) was determined as defined by Brown [1976]. Hemoglobin (Hb) concentration was measured according to^89^. The serum concentrations of alanine transaminase (ALT), aspartate transaminase (AST), creatinine, urea was measured using standard kits (Sigma-Aldrich, MAK080, MAK006, MAK052, MAK055, respectively). C-reactive protein (CRP) levels were assesed using commercial kit (AG723-M, Sigma-Aldrich).

#### Quantification of fecal Lipocalin-2

Fecal samples were collected from 1 to 7 day after colitis induction and dried at 37  °C in oven then PBS with 0.1% Tween 20 (100 mg/mL) was added and vortexed for 20 min to obtain a homogeneous fecal suspension. After that fecal suspension was microcentrifuged for 10 min at 4  °C, and levels of Lcn-2 were detected in supernatants using a Lipocalin-2 (Lcn-2) rat ELISA kit (ERLCN2, Thermo Fisher Scientific).

#### Enzyme linked immunosorbent assay (ELISA) for inflammatory cytokines

For assessing the levels of TNF-α, *IFN-γ* IL-6 and IL-10, colon tissues from different treatment groups were washed with PBS containing penicillin/streptomycin and then cut to 1-cm longitudinal sections. In the next step, serum-free RPMI 1640 medium with penicillin/streptomycin was used for colon sections culturing for 24 h, after that cell-free supernatants were collected and examined for cytokine secretion using Thermo Fisher cytokine ELISA kits (BMS622, BMS621, BMS625 and BMS629, respectively).

#### Assessment of colonic myeloperoxidase and nitric oxide

Myeloperoxidase (MPO) activity in the colonic tissue was measured as was defined by^90^. Colonic samples were weighed and homogenized in potassium phosphate buffers (10 mL), pH 6.0, hexadecyl trimethyl ammonium bromide, and ethylene acetic acid. In the next step, the homogenates were centrifuged for 20 min at 2000 rpm and the final supernatant was used for MPO assay by BioVision kits (E4581-100) and MPO results were expressed as units/g U/g of tissue wet weight.

Total NO in supernatant of colon homogenates was determined according to^91^ with slight modification of substituting zinc sulfate as an alternative of ethanol for the proteins precipitation. Absorbance was assessed at 540 nm and the data were quantified as nmol/g colonic tissue.

#### Assessment of lipid peroxides concentration and total antioxidant capacity

Detection of lipid peroxide levels, reflected as malondialdehyde (MDA), were done corresponding to thiobarbituric acid assay of^92^. The absorbance was estimated spectrophotometrically at 535 nm and the results were expressed as nmol/g tissue. Total antioxidant capacity (TAC) was measured by Cayman TAC assay kits according to manufacture guidelines. The content of the oxidized product was assessed at 405 nm. The samples' antioxidants capacity to prevent ABTS (3-ethylbenzthiazoline sulphonate) oxidation was matched to that of water-soluble tocopherol analogue (Trolox) and the results were measured as mmol of Trolox equivalent/g tissue.

#### RNA extraction and quantitative real-time PCR

Colon tissues were used for evaluating the expression of genes encoding cytokines [interleukin (IL)-1β, IL-6, IL-10, tumor necrosis factor α (TNFα), transforming growth factor-beta (TGF-β), and other inflammatory markers [cyclooxygenase-2 (COX-2) and Inducible nitric oxide synthase (iNOS)]. Besides, B cell lymphoma-2 (Bcl-2) and Bcl-2 associated x protein (Bax) as anti-apoptotic genes and pro-apoptotic genes as cytochrome c and caspase-3 were also, evaluated in colonic tissues. Total RNA was isolated from the collected tissues using RNeasy Mini kits (Qiagen, CA, USA) following the protocol's guidance. The purity and quantity of obtained RNA were verified using a NanoDrop™ ND-1000 Spectrophotometer (Thermo Fisher Scientific Inc., Waltham, MA, USA). Extracted RNA was reverse transcribed into cDNA by a Maxima First-Strand cDNA Synthesis kit (Thermo Scientific, USA). Synthesis of cDNA was done at 60-min incubation at 37  °C for activation then heating to 95  °C for 5 min, and ultimately, a holding temperature of 4  °C (thermal cycler with Averiti 96-well, Applied Biosystems, Foster City, CA, USA). The expression analysis of inflammation and apoptosis-related target genes by quantitative real time PCR (qRT-PCR) using SYBR green Kit (Qiagen, Hilden, Germany) and specific primers for each target gene. All PCR reactions were completed, in triplicate, in the Stratagene MX3005P real-time PCR machine (Stratagene, La Jolla, CA, USA). The relative genes expression levels were calculated using 2–ΔΔCT method^93^, where β-actin and was used as a housekeeping gene. The sequences of genes specific primer are presented in Table 4.

**Table 4:** Primer sequences utilized for rRT-PCR analysis of targeted gene expression.

Target gene	Primer sequence (5′–3′)	Accession No./Reference
iNOS	F-ACCTTCCGGGCAGCCTGTGA R-CAAGGAGGGTGGTGCGGCTG-3′	NM_012611
*COX-2*	F-GCTCAGCC ATACAGCAAATCC R-GGGAGTCGGGCAAT CATCAG	NM_017232
*Caspase-3*	F-GCAGCTAACCTCAGAGAGACATTC R-ACGAGTAAGGTCATTTTTATTCCTGACTT	NM_012922
*Bcl-2*	F-TGCGCTCAGCCCTGTG R-GGTAGCGACGAGAGAAGTCATC	NM_016993
*BAX*	F-CAAGAAGCTGAGCGAGTGTCT R-CAATCATCCTCTGCAGCTCCATATT	NM_017059
*Cytochrome C*	F-TTTGAATTCCTCATTAGTAGCTTTTTTGG R-CCATCCCTACGCATCCTTTAC	NM_012839
*IL-1β*	F-TGACAGACCCCAAAAGATTAAGG R-CTCATCTGGACAGCCCAAGTC	NM_031512.2
*IL-6*	F-CCACCAGGAACGAAAGTCAAC R-TTGCGGAGAGAAACTTCATAGCT	NM_012589.2
*IL-10*	F-GCCCAGAAATCAAGGAGCATT R-CAGCTGTATCCAGAGGGTCTTCA	L02926.1
*TNFα*	F-CAGCCGATTTGCCATTTCA R-AGGGCTCTTGATGGCAGAGA	L19123.1
*TGF-β*	F-CCAGCCGCGGGACTCT R-TTCCGTTTCACCAGCTCCAT	NM_021578.2
β-actin	F-CGCAGTTGGTTGGAGCAAA R-ACAATCAAAGTCCTCAGCCACAT	V01217.1
*GAPDH*	F-TGCTGGTGCTGAGTATGTCG-3′ R-TTGAGAGCAATGCCAGCC-3′	NM_017008
*Bacteroides spp.*	F:GAG AGG AAG GTC CCC CAC R:CGC TAC TTG GCT GGT TCA G	Layton et al.^94^
*Firmicutes spp.* Guo et al. 2008	F-GGAGYATGTGGTTTAATTCGAAGCA R-AGCTGACGACAACCATGCAC	Guo et al.^95^
*Enterobacteriaceae*	F-CATTGACGTTACCCGCAGAAGAAGC R-CTCTACGAGACTCAAGCTTGC	Bartosch et al.^96^
*Bifidobacterium spp.*	F-GCG TCC GCT GTG GGC R-CTT CTC CGG CATGGT GTTG	Requena et al.^97^
*Bacillus spp.*	F-GCA ACG AGC GCA ACC CTT GA R-TCA TCC CCA CCT TCC TCC GGT	Zhang et al.^37^

*iNOS* inducible nitric oxide synthase, *COX-2* cyclo-oxygenase-2, *IL* interleukin, *TNFα* tumor necrosis factor α, *TGF-β* transforming growth factor-beta, *COX-2* cyclooxygenase-2, *Bcl-2* B cell lymphoma-2, *Bax* Bcl-2 associated x protein.

#### Quantitative DNA-based analysis of abundance of colon bacterial populations

Total DNA was extracted from colon contents using QIAamp Fast DNA Stool Mini (Qiagen, Hilden, Germany). The quality and concentration of extracted DNA were evaluated by a Thermo Scientific NanoDrop 2000 spectrophotometer (Thermo Fisher Scientific Inc., USA). Samples of purified DNA were stored at − 80 °C for latter quantitative PCR assessment. Real time PCR (RT-PCR) were completed to calculate the abundance of selected bacterial spp including *Bacillus*, *Bacteroides*, *Firmicutes*, *Enterobacteriaceae,* and *Bifidobacterium*, using Stratagene MX3005P quantitative PCR. The primers sequences targeting bacterial specific genes are presented in Table 4. The PCR amplification analyses were conducted, in triplicate, in 25 μL reaction containing 1 μL of of each primer (10 mM), 12.5 μL of SYBR Green PCR Master Mix (Qiagen, Germany), 9.5 μL of sterile PCR grade water and 2 μL of specific genomic DNA. Standard curves were prepared with ten-fold serial dilutions of genomic DNA isolated from pure bacterial cultures. Then standard calibration curves were made by plotting the threshold cycle (Ct) values vs. the bacterial DNA copy numbers. The bacterial concentrations in each DNA sample were quantified, by the produced standard curves, in terms of log_10_ CFU/gram of the colon contents.

#### Histopathological analysis

Tissue samples from the distal colon were fixed in 10% neutral buffered formalin for 24 h then were dehydrated in grade ethanol, embedded in paraffin and cut using microtome (Leica). Then, obtained tissue sections (5-μm) were processed for staining using standard H&E procedure and examined under light microscopy^98^. The histological examiner looked for lesions (such as, distorted crypt architecture, epithelial damage, and inflammatory cells infiltration^99^.

#### Immunohistochemical detection of Ki67 and IL-6.

The immunohistochemical procedure was done as previously reported by^82,100^. In brief, paraffin embedded tissue sections (5-μm) were rehydrated in xylene and then in graded ethanol concentrations and heated in citrate buffer (pH 6) for 10 min in microwave for antigen retrieval. Then, blocking of nonspecific binding was done using blocking buffer (3% bovine serum albumin in Tris-buffered saline or TBS) for 1 h at room temperature. The sections were incubated overnight at 4 °C with the primary antibody; anti-NF-kB p65 (1:250, ab16502, abcam) or anti-caspase-3 (1 µg/mL, ab184787, abcam). Blocking of endogenous peroxidase was done by incubating the slides with 0.3% H_2_O_2_ in TBS for 15 min. Following primary antibody incubation step, the slides were washed with TBS and were then incubated with goat anti-rabbit HRP secondary antibody (1:1000, ab205718, abcam) for 60 min at room temperature in in a humidified slides chamber. Finally, the slides were washed with TBS and the positive reactions were detected using the DAB chromogenic agent (Expose mouse and rabbit specific HRP/DAB detection kit, ab80436, abcam). Negative control slides were obtained by the same steps without using the primary antibody. The cells’ nuclei were counterstained with Mayer’s hematoxylin. The immunostained sections were examined with light microscopy and the intensity of staining was measured using imageJ software (v 1.53, National Institutes of Health, Bethesda, MD, USA) as previously reported^101^.

### Statistical analysis

Statistical comparisons of the results were done using SPSS^®^ Statistics program version 22 (SPSS Inc., USA). The normality of variance and homogeneity were assessed by Shapiro–Wilk and Levene tests, respectively. The data were analyzed by one-way analysis of variance (ANOVA) then differences among groups were assessed by Tukey-post hoc. All variables were expressed as means ± standard errors (SE). Statistically significant level was considered at P values  < 0.05. Differences were esteemed significant when P < 0.05. All graphs were created using the GraphPad Prism software (Version 8, GraphPad Software Inc.).

## Data Availability

The datasets generated and/or analysed during the current study are available from the corresponding author on reasonable request.
